# The Change in Hospital Finance
*Read at the Twelfth Conference of the British Hospitals Association, May 25.


**Published:** 1922-06

**Authors:** Alan Garrett Anderson


### THE CHANGE IN HOSPITAL FINANCE.*

By SIR ALAN GARRETT ANDERSON, K.B.E.

The change which has taken place in the finance of Hospitals and their need for union in the British Hospitals Association in order that they may meet the change.

YOU will all have noticed that differences of opinion in hospital politics may be clothed in academic language, but they are quite as sharp as differences in politics elsewhere, and so, as I am an official of King Edward's Hospital Fund, which has been appointed as the Voluntary Hospitals Committee of the London Area, I must warn you that the views which I express are mine as an individual, and are not necessarily the official policy of the Fund.

I must start by asking for mercy as a non-expert addressing experts, but in one sense the narrow frontiers of my knowledge are a help. There is only one piece of hospital work about which I am at all qualified to speak, and when I was asked to address you I felt sure that my invitation was to say something on this text—the change in the finance of hospitals.

I need not argue whether or not finance of hospitals is in the melting pot. This is common knowledge throughout the United Kingdom, and it would be a miracle if, when all other finance is in a state of flux, hospitals alone could make their old revenue square with their new expenses. For hospitals more than most other enterprises the need for change is acute, for even before the financial revolution of the war the established system of hospital finance was beginning to grow old and tired, and why not? Since hospitals first began to beg from the rich in order to give free treatment for the poor, the industrial and social structure of the country has completely changed, and it would be a miracle if the system devised for one age proved to be the best for a new and different age. So long as the community was sharply divided into rich and poor, so long as those who go to the hospitals could be described as "the sick poor," so long the simple rule of finance by begging was perhaps sufficient, but immediately the community realised that working men and women wanted to be independent, ought to be independent, and must be paid by industry to pay their way as free citizens, then immediately change or reform in hospital finance began to be overdue, and with every year that passed and every victory of the workers in their long struggle for better conditions, the anomaly of treating them gratuitously in hospital, all lumped together as the sick poor, became more acute and the strain on the hospital budget more intolerable.

I have been looking through the addresses to which you have listened at your various annual meetings, and I find that one after another, starting with Mr. C. S. Lock at your very first meeting in 1910, the speakers pointed out the dangers of the free treatment of patients in hospital. When Sir William Osler addressed you in 1913, he spoke of this system of finance by begging, calling it "the voluntary system." I do not at all agree that charity finance is the essence of the voluntary system, but what Sir William Osler said was "My advice is very brief and direct—give it up. It is very antiquated, out of date, and is not going to continue. Make up your minds that you must accept the principle of taking pay from patients, it answers admirably elsewhere."

Now we all know that when a change is pressed upon us we can either accept it, and say with the Romans, "times change and we change with them," or we can resent the change and complain that the times are out of joint; which of the two attitudes is likely to lead to the better results? In surgery and medicine hospitals are not slow to change. They are the pioneers, the leaders of progress. Why then in finance should hospitals shrink from adding to their armoury a weapon which has proved its worth all over the world—the weapon of appeal—"*Be independent.*" There are several reasons which endear the old charity finance to the hospitals, but one sufficient cause for their finding it difficult to change even after they have made up their minds that they must change, and that quickly, is the central text of my remarks to you.

The hospitals of Great Britain were slow to move because they were not in close enough contact with one another to adopt a common policy, and it is because it seems to me that, in the time of change and crisis, it is essential that the hospitals as a whole should be able to think and act for their common interest with reasonable speed that I welcome the chance of coming to this meeting of the British Hospitals Association to make my confession of faith.

Let us glance for a moment at some of the results of the failure of hospitals to move with the times in these last 20 years. Every hospital has been encouraged to expose its sores in order to get charity, and naturally, as charity was apt to go to the biggest debts, there was little inducement to avoid them. Among cases of equal urgency the hospitals themselves made poverty the passport for treatment, and as the public gave them little encouragement for thrift, they gave little encouragement to their patients to provide for a rainy day when they would need the help of hospitals.

Again, the finance of this great and necessary work by charity has been a heavy strain upon the treasurers of the hospitals, and upon the comparatively small section of the community from which they have begged, and as a result the work of the hospitals has been cramped by poverty and the patients have, in many cases, to suffer delay or discomfort, which they would have gladly paid to avoid. These defects are only the result of a system which rewards poverty rather than efficiency. In London, and I have no doubt elsewhere, a constant struggle goes on to

*Read at the Twelfth Conference of the British Hospitals Association, May 25.

correct these inevitable defects, but the only cure is a radical change.

Perhaps I have said enough on the need for change, especially as it has been the theme of addresses to you since your first meeting, but I must also say something about its dangers. Charity finance gives hospitals the freedom from control and interference and criticism, which is very attractive. On the other hand, finance by patients or prospective patients presupposes a large and active body of opinion, quite disposed to criticise, and to take a hand in hospital management, which is good, but also, alas, slow to draw the line between mundane affairs on which the layman may properly speak, and questions of scientific and medical interest, on which medical men must claim to be supreme. Again, if we admit the principle that everyone ought to pay for himself in hospital according to his means, can hospitals continue to relieve the indigent ? Can we preserve the glory of the old system and still keep in step with society around us ?

As my work lies in London, it is London I must quote, and I do so diffidently because it is not London that has given the lead in this change—the road we think we must follow has been explored, and pointed out to us in the North and in the Midlands. Perhaps I may expand a little on this theme for those who, like myself, come from the South, and may not know what progress has been made elsewhere. I will give you one instance. The Midland Railway, on whose Board I sit, has received from its men—from a committee representing about 60,000 men, of whom about 6,000 are in London—a request to join with them in a regular contribution to hospitals, sufficient to pay the full maintenance cost in hospital of the men, their wives, and families ; the men asked the Company to bear one-third of the cost as a working expense, and to deduct two-thirds from their wages ; the proposal was examined in detail by the Board, and at our last annual meeting our chairman, with the unanimous assent of his colleagues, announced that the Board had approved in principle the proposal of the men, and that he would in due course inform the shareholders to what extent he considered they should supplement the men's contributions.

In these discussions we learned that in certain railway and other shops in Derby, over 90 per cent. of the men had already started a regular contribution to the local hospital of 1d. per week, and that as this was found insufficient they had raised their subscription to 2d. per week. In Sheffield we were told that many of the large firms joined with their men in the contribution at the rate of 1d. in the £ of annual wages, and in other districts the same idea is being put in force in various shapes.

Now, there is nothing peculiar in the case of the Midland Railway ; the men of other railways have the same need of hospitals, the same spirit of independence, the same tenderness for wife and child—the directors of all our large businesses have the same desire to help their men to help themselves, and the same belief that private enterprise in hospitals—as in transport—will make rings round a Government department every time. The experience of Sheffield and other large cities confirms this example I have given. Need we doubt the response of industry—men and employers—when the hospitals invite its support for a well-designed and national contributory scheme ?

In London the great majority of the hospitals have decided to tell their patients that an independent working man ought to provide for himself and his dependents in sickness as he does in health, and to organise a system under which that provision can be made. They are setting up a company with limited liability, but with no power to make profits, called the Hospital Saving Association, which will invite persons within the hospital income limits to subscribe week by week or year by year the premium appropriate to cover their risk ; on this Association the hospitals, the central funds and the contributors will be represented. The H.S.A. will either pay to hospitals which treat contributors to the H.S.A. the charge, if any, which they expect from their patients, or by arrangement it may prove possible to distribute the income of the Hospital Saving Association among the co-operating hospitals, on some principle other than the precise number of contributors treated in the year.

To avoid complications I will assume that at first all payments to hospital will be made in respect of contributors to the H.S.A. actually treated, and will be at least as much as the hospital charges to other patients. We calculate that a premium of 3d. per week, or 12s. per annum, paid in advance by a working man to cover the risk for himself, his wife or dependent children, would work out as follows :—

| | |
|---|---|
| 100 contributors at 12s., pay to H.S.A. per annum ... ... ... ... ... | £60 |
| Of these *two* become in-patients for three weeks, each at £4 cost to hospital per week ... | £24 |
| Leaving a balance of ... ... ... ... ... | £36 |

to cover out-patient attendance on the 100, and the whole cost of their wives and dependent children.

It is neither necessary nor possible to attempt any exact estimate—not necessary, because this contribution of 12s. per annum is certainly far more than we get at present ; not possible because, when the hospital finance is revised and when the income depends upon the work done by hospital rather than upon its debt, it may be found good both for hospitals and community to extend the hospital services, and the effect may well be to vary the incidence of in-patient sickness, upon which any estimates of cost must, of course, be based.

The chief criticism raised in London against trying any system of contributory finance was that it implied or involved a contract of treatment, and that hospitals, and particularly teaching hospitals, must keep themselves quite free to reject or receive patients on medical grounds alone. The Hospital Savings Association is not open to this criticism, as each hospital will continue to select its patients exactly as it has always done, and need not know that the patient is a contributor to the Hospital Saving Association till the time comes for payment. The hospitals will probably find it desirable to hold their almoner's inquiries into means at the time when the individual becomes a contributor to the Hospital Saving Association, and after this is done it will not be necessary to hold another inquiry when the patient reaches the hospital. I understand that the contributors will set store by this change, as they dislike inquiry about their means when they reach the hospital, but, of course, everyone is agreed that the almoners' inquiries as to means are absolutely necessary in every case in which the patient asks for any remission of charges, either from the hospital or from the doctor. As a part of this system the hospitals will no doubt combine together in the appointment of almoners, so that in each workshop where the employees become contributors to the Hospital Saving Association, one almoner can conduct all the inquiries as to means on behalf of all the hospitals.

The Hospital Saving Association will ask employers to join their employees, and to contribute one-third of the total contributions. In such cases where masters and men join, the collections will be made by the masters in the pay-roll. In passing I should say that this system will promote the financial claims of hospitals from their present humble position as the last item on the industrial budget to the position they ought to occupy, as one of the working costs of an organised business community. The employer knows he has to pay his men enough to buy food and clothes for their wives, children and themselves, so apart from the occasional dispute about wages the working man's income adjusts itself automatically to meet changes of cost of living. The cost of maintenance during sickness should have the same claim as maintenance in health, and ought to be given the same priority over profits and over the claim of the income tax collector.

It would, of course, be easy to include in this scheme persons outside ordinary hospital income limits, if the hospitals were prepared to treat wealthier patients, but the present intention is to ask for a contribution calculated to cover the cost of maintenance of an ordinary hospital patient, and not the extra charge which hospitals might properly expect for themselves or their staff from a rich patient.

We hope that in London the Hospital Saturday Fund will collect for and work closely with the Hospital Saving Association, and that a subscription by employers and men to the H.S.A. will gradually become the mark of a well-organised trade and a well-managed office. When that point is reached, and, of course, in an immense city such as London, it will take years of work to arrive there, we shall find the hospitals treating the same class that they treat at present, but that a large fraction of the patients pay their own way, and set free for the indigent the charity given for "the sick poor," but at present devoted to wage-earners who ought to provide for themselves.

In 1920 the expenses of the London hospitals exceeded the income by 14 per cent; that is the gap we have to bridge at once. We may hope to do more later on, but we want this at once. It is no use to increase one part of our income at an equivalent loss elsewhere. We must maintain our income from charity—which was, in 1920, 50 per cent. of the whole—and our income from other sources.

Throughout London, and I suppose throughout England, the patient's payments tend to increase; the tendency must be accelerated, and the H.S.A. will very materially assist, as each contributor who becomes a patient will have saved enough to pay his way.

Some of you have for years worked similar schemes; they differ between themselves in almost every particular, but they agree in one, and that the chief point. One and all, these schemes of contributory finance by prospective patients have enormously increased the income of hospitals, but they have disclosed difficulties and dangers or at least problems which will affect all hospitals as they travel along the same road.

The contributor wants representation; in fact, unless he gets it, unless he feels that the hospitals are "his hospitals," he won't contribute, so this problem must be solved at the start; but you will all agree that it is not happily solved if the hospital becomes the tied house of a section of the community. The contributors must be able to make themselves heard, but they are not the only pebble on the beach.

Next, the help the hospital gets from its patients must not scare away charity, or we shall have done more harm than good, and we all know that it is easy to frighten away donations. But in this we are between the devil and the deep sea. If we don't get more income the hospitals will smash; the deficiency is 14 per cent. only, but water an inch above his head is deep enough to drown a man, and if once the charitable public gets the idea that the voluntary system can't be saved by its subscriptions, the State will be forced to take over hospitals as it has taken over schools; voluntary hospitals will no more be able to command general public support than voluntary schools do to-day.

It is well to remember also that if the State is forced to take over the weaker hospitals, the impression that the voluntary system is doomed will decrease subscriptions and endanger even the wealthiest hospital. This is no time for selfishness—the hospitals must sink or swim together, and so in London all but one of the large hospitals and almost all the small hospitals are united for the first time in a Combined Appeal for money to clear their feet for a time until they can start the contributory system about which I have told you, and can increase in other ways the support they get from those who use the hospitals.

So much for the need for change, and what we are doing in London to meet it. There remains the organisation.

We at the King's Fund, and here I give you the firm and unanimous opinion of the whole Council of the Fund, are convinced that the individuality and freedom of the hospitals must be maintained. Voluntary hospitals—in the sense of hospitals organised and controlled by private enterprise—seem to us far more likely to give the nation the medical education, the research, the hospital services, that it needs than any State-controlled service. We don't want another great public department in charge of the hospitals, and whether we speak as tax-payers or as occasional patients of our family doctor, we don't want the State to organise and engage doctors on an annual salary.

Our objections to bureaucratic control are not decreased if we think we might ourselves become the Government department in charge of the hospitals, and the King's Fund, as the Voluntary Hospitals Committee for London, is at present an agent of the Government. In all our dealings with the hospitals we have therefore tried, even at the risk of delay, to secure decision and action by the hospitals rather than to force them along a line selected by us.

If the Voluntary Hospitals are to endure, the initiative must be theirs, and it is just at a time like this—a time of crisis—that they must show their power to think and act together. In these difficult and sometimes heated negotiations we should have failed over and over again, if it had not been that the hospitals of London, through the Regional Committee of the British Hospitals Association, applied themselves to this new task with the spirit which has made them what they are to-day, and asked us, the King's Fund, with a practically unanimous voice, to lead them in a policy they themselves had laid down.

I submit to you that it rests with the hospitals themselves, and as they must act together, it rests most of all with the Regional Committees of the British Hospitals Association to save the voluntary principle of hospital administration.

---

### HOSPITAL DIETITIANS.

### THEIR TRAINING IN AMERICA.

AMERICAN hospitals have lately made a practice of appointing dietitians, and therefore the following summary of their training and qualifications may be of interest. It is taken from an article by Miss Octavia Hill, head dietitian at Peter Brent Brigham Hospital, Boston, in "The Modern Hospital."

The candidate, says the writer, should preferably be a college woman of 25 with a B.S. degree and "a major in home economics." Height, smartness of address and, in appearance, a professional attitude and humour, are personal advantages. On arrival at the hospital, the first month of training is spent in the main kitchen, where each worker's separate duty is studied in turn. The student then passes to the serving room, from which 300 persons and four dining-rooms are supplied. The service of food, like the preparation of a meal, is here studied in detail. The care of dining-rooms, crockery, and the appearance of tables and of the helpings are taught, since untidiness, too large helpings and poor waiting destroy the attractiveness of a meal. On stated days, in the matron's absence, the student supervises one or other of these departments. A week in the infants' department follows, where the computation and preparation of special diets are taught. The weights of food are particularly studied, so that the number of grammes in, say, a salad or a moderate helping of meat is learnt. Special diets, such as nephritic or diabetic patients require, are next explained. Popular and unpopular foods are tabulated and patients' reaction to diet observed. The ward kitchens are visited at meal-times to see that the service has not affected the dishes on the way. Seven weeks are spent in the diet kitchen. Here the student learns how the weekly and daily supplies are ordered, their cost, and the weekly cost per head. A chart is kept of the rise or fall of this expenditure. Another week is spent in the dietitian's office, where the department's accounts are studied, and the store visited. The checking of supplies is explained. A visit is paid to the market with the dietitian and the superintendent, but Miss Hill says that experience and not observation is the sole guide to buying. The writing of bills of fare for every class in the institution is next taught, and the amounts to be requisitioned from the store for each person are gone into. Every week a conference with the students is held, when daily problems and the instruction of nurses in dietetics and cookery are discussed. A month may be added in the "metabolism laboratory" and a nutrition clinic attended. The hospital does not depend upon the work of these students, twenty-six of whom have taken the course in the past two and a half years.

---

### MEDICAL TRAINING OF THE MISSIONARY.

AN institution which deserves to be much better known is Livingstone College, where the elements of medicine are taught to missionaries. The value of this work is so great to the missioners, the societies under which they work, and the distant peoples with whom they live, that we have often said that a medical training of this kind should be an essential qualification for all entering the field of foreign missions. The full course lasts for a year and costs about £100, but shorter courses are also given and appreciated, including a vacation course in July for transatlantic visitors passing through England on their way to their destinations. Last year, for the first time, the courses were thrown open to women, five of whom enrolled. The latest report is extremely interesting, and Sir George Makins' testimony to the value of the medical training given is decisive. It seems almost irrelevant to add that the students sign an agreement not to call themselves medical missionaries; and as Sir George remarks, cases of very severe injury and disease are he exception. The students have the benefit of practical clinical teaching at Poplar Hospital and at St. James-the-Less Medical Mission, Bethnal Green. The need of the moment is to found some bursaries of, say, £50 in value, to be offered to the mission societies or to suitable candidates. It is still doubtful whether the societies make the most of the advantages of the College. At present we believe that even the instruction of the missioners in personal health is not the rule among all the societies.

